# Development and field validation of a large-volume bag-based whole-blood collection method in donor dairy cows

**DOI:** 10.3389/fvets.2026.1906488

**Published:** 2026-08-19

**Authors:** Filippo Rigo, Luca Panozzo, Giacomo Catarin, Margherita Soncin, Antonio Barberio, Flaviana Gottardo

**Affiliations:** 1 Department of Animal Medicine, Production and Health, University of Padua, Padua, Italy; 2 Istituto Zooprofilattico Sperimentale delle Venezie, Padua, Italy

**Keywords:** bacteriological contamination, blood bag, blood-derived products, cattle, large-volume blood collection, platelet-derived products, whole-blood collection

## Abstract

Obtaining sterile anticoagulated whole-blood units is a key prerequisite for downstream laboratory and clinical applications involving blood-derived products. In bovine medicine, however, published information on blood collection into bags is mainly embedded in transfusion-oriented references or broader downstream studies, while formal validation of donor-oriented collection methods remains limited. The aim of this study was to develop and field-validate a large-volume bag-based method for whole-blood collection in donor dairy cows under commercial farm conditions. Whole-blood collections were performed in clinically healthy donor dairy cows using a jugular venipuncture protocol that was progressively refined during field validation. A true sequential multi-bag setup, enabled by extension tubing and a perforable connection device, was introduced on 24 October 2022. Protocol validation was based primarily on procedural success, defined as a bacteriologically negative blood unit with a collected volume of at least 400 mL per bag. A total of 294 procedures were included. Overall, 228/294 procedures (77.6%) were classified as successful, 48/294 (16.3%) as suboptimal, and 18/294 (6.1%) as failed. The median collected volume per procedure was 410 mL (IQR 400–430; range 124.5–1,280 mL). Overall, 276/294 collections (93.9%) were bacteriologically negative. Within the single-bag phase, later collection dates were associated with a lower probability of bacteriological positivity, supporting a learning-curve effect on microbiological reliability. Later implementation of the sequential multi-bag setup was associated with a higher total volume per procedure, without an apparent loss of microbiological safety. In conclusion, this large-volume bag-based protocol proved feasible for whole-blood collection in donor dairy cows under field conditions and may provide a practical upstream basis for subsequent laboratory applications and downstream processing workflows.

## 1 Introduction

Obtaining sterile anticoagulated whole-blood units is a fundamental prerequisite for several clinical and laboratory applications in cattle, including blood transfusion and the preparation of blood-derived products intended for subsequent processing. For the production of platelet-derived products, whole blood should be collected into anticoagulant-containing systems to prevent clot formation and preserve platelet integrity and processability during subsequent component separation (1). In addition, maintaining sterility at the whole-blood collection stage is essential because contamination of the collected unit may compromise subsequent handling, processing, and final product usability (2). In this context, the blood bag should be regarded not merely as a collection device, but as a means of obtaining anticoagulated and microbiologically suitable whole blood for downstream processing.

In bovine medicine, whole-blood collection into bags has been described mainly in the context of transfusion practice and blood-handling studies, and practical recommendations for donor selection and blood use are available in the ruminant literature (3, 4). Bovine whole blood collected in anticoagulant-containing plastic bags has also been investigated under field and storage conditions, including following pre-storage leukoreduction, confirming the practical feasibility of bag-based collection in this species (5). However, most of the available literature is primarily oriented toward transfusion use, blood conservation, or downstream applications rather than toward the formal development and field validation of a donor-oriented large-volume collection method under commercial farm conditions. As a result, practical aspects such as achievable collection volume, preservation of sterility, maintenance of a closed collection system, and procedural reproducibility under routine farm conditions remain insufficiently defined for this specific purpose.

Interest in blood-derived and platelet-based products has increased in veterinary medicine because of their potential applications in tissue repair, inflammatory modulation, and regenerative medicine. At the same time, the available literature highlights substantial heterogeneity in preparation methods and limited standardization across both upstream and downstream phases, including donor selection, blood collection, sample handling, and product manufacturing (6–8). This methodological variability remains a major obstacle to reproducibility and comparability among studies. The issue is also relevant in cattle, where platelet concentrates and related hemoderivatives have been investigated for mammary and other clinical conditions. In particular, bovine mammary applications have attracted increasing attention, confirming the practical interest of blood-derived products in dairy medicine while also emphasizing the need for robust and reproducible collection and processing workflows (8–10). Moreover, donor-related studies in cows have highlighted the importance of animal selection and baseline donor characteristics for subsequent blood component yield, further supporting the need to standardize the collection phase itself (11).

In this context, the availability of a practical and reliable field method for collecting sterile, anticoagulated, large-volume whole-blood units may represent a key prerequisite for the development of blood-derived product workflows in bovine medicine. If donor blood collection remains technically difficult, poorly standardized, or highly operator-dependent under field conditions, it may limit the broader applicability of downstream hemocomponent- and hemoderivative-based approaches. In the present study, the term “large-volume” is used in an operational sense to indicate collection procedures aimed at obtaining at least one adequately filled blood bag and, when feasible, the sequential collection of additional bags within the same procedure. It therefore refers to practical collection output under field conditions rather than to a fixed proportion of total circulating blood volume.

The present protocol was developed within a broader project requiring repeated whole-blood collection from donor dairy cows under commercial farm conditions. Several methodological challenges emerged during protocol development, including the need to obtain relatively large volumes through jugular collection, preserve microbiological sterility, and progressively stabilize an operator-dependent field technique. Although blood collection into anticoagulant-containing bags is feasible in cattle and has been described in related contexts, a dedicated report focused on the development and field validation of a donor-oriented large-volume protocol was still warranted.

Therefore, the aim of the present study was to develop and field-validate a large-volume bag-based whole-blood collection method in donor dairy cows, with particular emphasis on procedural feasibility, collected volume, and bacteriological safety for subsequent downstream processing.

## 2 Materials and equipment

The main materials, devices, and equipment required for the large-volume whole-blood collection protocol are summarized in Table 1.

**Table 1:** Materials and equipment required for the large-volume whole-blood collection protocol in donor dairy cows.

**Item**	**Specification**	**Purpose**	**Protocol phase**
Donor animals	Clinically healthy donor dairy cows	Source of whole blood for collection	Single-bag and multi-bag phases
Restraint facilities	Field-appropriate cattle restraint system	Safe standing restraint during jugular venipuncture	Single-bag and multi-bag phases
Blood collection bag	Sterile blood collection bag prefilled with CPDA-1, equipped with integrated tubing and collection needle	Collection of anticoagulated whole blood in a closed sterile system	Single-bag and multi-bag phases
16-gauge cannula needle	16-gauge	Jugular venipuncture and blood flow establishment	Implemented multi-bag phase only
Extension tubing	Compatible with blood collection bag system	Sequential connection of an additional bag during the implemented multi-bag phase	Implemented multi-bag phase only
Perforable connection device	Compatible with the blood collection bag system	Connection of an additional collection bag while maintaining system integrity	Implemented multi-bag phase only
Clippers or razor	Standard field-use device	Hair removal at the venipuncture site when needed	Single-bag and multi-bag phases
Disposable gloves	Clean or sterile, as appropriate	Aseptic handling during site preparation and collection	Single-bag and multi-bag phases
Povidone-iodine tincture	Betadine	Antiseptic preparation of the venipuncture site	Single-bag and multi-bag phases
Ethanol-soaked gauze	Gauze soaked in 90% ethanol	Antiseptic preparation of the venipuncture site	Single-bag and multi-bag phases
Labels or tracking identifiers	Standard identification system	Traceability of collected units	Single-bag and multi-bag phases
Clean or insulated transport container	Suitable container for field transport of collected units	Preservation of unit integrity after collection	Single-bag and multi-bag phases
Sterile sampling materials	Sterile syringes or sampling devices	Post-collection sampling of blood units	Single-bag and multi-bag phases
Sterile sample tubes or containers	Standard sterile collection consumables	Collection of post-collection samples for bacteriological testing	Single-bag and multi-bag phases
Microbiological media and laboratory consumables	Routine laboratory materials	Laboratory bacteriological examination of collected samples	Single-bag and multi-bag phases
Weighing system	Portable digital scale	Monitoring of collected blood volume	Single-bag and multi-bag phases
Refrigerated or insulated transport devices	When required by the workflow	Maintenance of sample integrity during transport	Single-bag and multi-bag phases

The table summarizes the main materials, devices, and equipment used for donor handling, blood collection, site antisepsis, post-collection handling, and bacteriological assessment. The original protocol was based on a standard single-bag blood collection system. During the implemented multi-bag phase, a 16-gauge cannula needle, extension tubing, and a perforable connection device were added to enable true sequential multi-bag collection within the same procedure.

### 2.1 Donor animals

Clinically healthy donor dairy cows from commercial herds were considered for inclusion in the collection procedure. Animals were selected on the basis of general health status, absence of systemic illness, absence of ongoing pharmacological treatment, and suitability for jugular venipuncture under field conditions. Only animals considered clinically fit and manageable during restraint were included.

### 2.2 Blood collection bags and collection devices

Whole blood was initially collected using commercially available sterile blood collection bags prefilled with CPDA-1 anticoagulant solution. Each collection set consisted of a sterile collection bag equipped with integrated tubing and a dedicated collection needle. The initial configuration was based on a single-bag setup designed to obtain anticoagulated whole blood in a closed system.

During protocol refinement, additional devices were introduced to enable true sequential multi-bag collection within the same procedure. These included a 16-gauge cannula needle for jugular venipuncture, extension tubing, and a perforable connection device compatible with the blood collection bag system.

### 2.3 Materials for site preparation

Preparation of the venipuncture site was performed using materials for clipping, skin cleaning, and antisepsis. These included disposable gloves, clippers or razors for hair removal when needed, sterile gauze, 90% ethanol, and povidone-iodine tincture. Site antisepsis was performed using three consecutive passes of povidone-iodine tincture, followed by gauze soaked in 90% ethanol. Additional clean or sterile disposable materials were available to maintain aseptic handling during site preparation and blood collection.

### 2.4 Materials for post-collection handling and sampling

After collection, each bag was identified using a label or tracking identifier and placed in a clean, protected transport container. Transport conditions were selected to preserve sample integrity and minimize contamination and excessive heat exposure. When post-collection bacteriological testing was required, samples were collected using sterile syringes or sampling devices and transferred to sterile tubes or containers.

### 2.5 Laboratory materials and equipment for bacteriological assessment

Post-collection microbiological evaluation was performed in the laboratory. Bacteriological examination was carried out using routine microbiological media and standard laboratory equipment and consumables. The main equipment required for the overall workflow included field-appropriate restraint facilities for donor cows, a commercial digital scale for monitoring collected blood volume, and a clean or insulated transport container, when required by the workflow.

## 3 Methods

### 3.1 Study setting

Whole-blood collections were performed in commercial dairy herds within a broader project requiring repeated donor blood collection for subsequent laboratory use. All procedures were carried out on farm by veterinarians or trained veterinary personnel. During the study period, the collection procedure was progressively refined as part of the method development process, and true sequential multi-bag collection within the same procedure was introduced only in the later phase, beginning on 24 October 2022.

### 3.2 Protocol overview

Summary of protocol inputs, outputs, and operational characteristics:

- *Input*: clinically healthy donor dairy cows; sterile CPDA-1 blood collection bag system; field-appropriate restraint facilities; aseptic preparation materials; sterile sampling materials for bacteriological assessment when required.
- *Output*: sterile anticoagulated whole-blood unit(s) suitable for microbiological assessment and subsequent downstream processing.
- *Procedure duration*: variable according to donor handling, venous access, blood flow stability, and whether sequential multi-bag collection is attempted. Site preparation generally requires a few minutes, whereas the active collection phase is variable.
- *Required expertise*: veterinarians or trained veterinary personnel experienced in bovine restraint, jugular venipuncture, aseptic handling, and blood bag collection procedures under field conditions.

### 3.3 Step-by-step procedure

**Figure 1:**
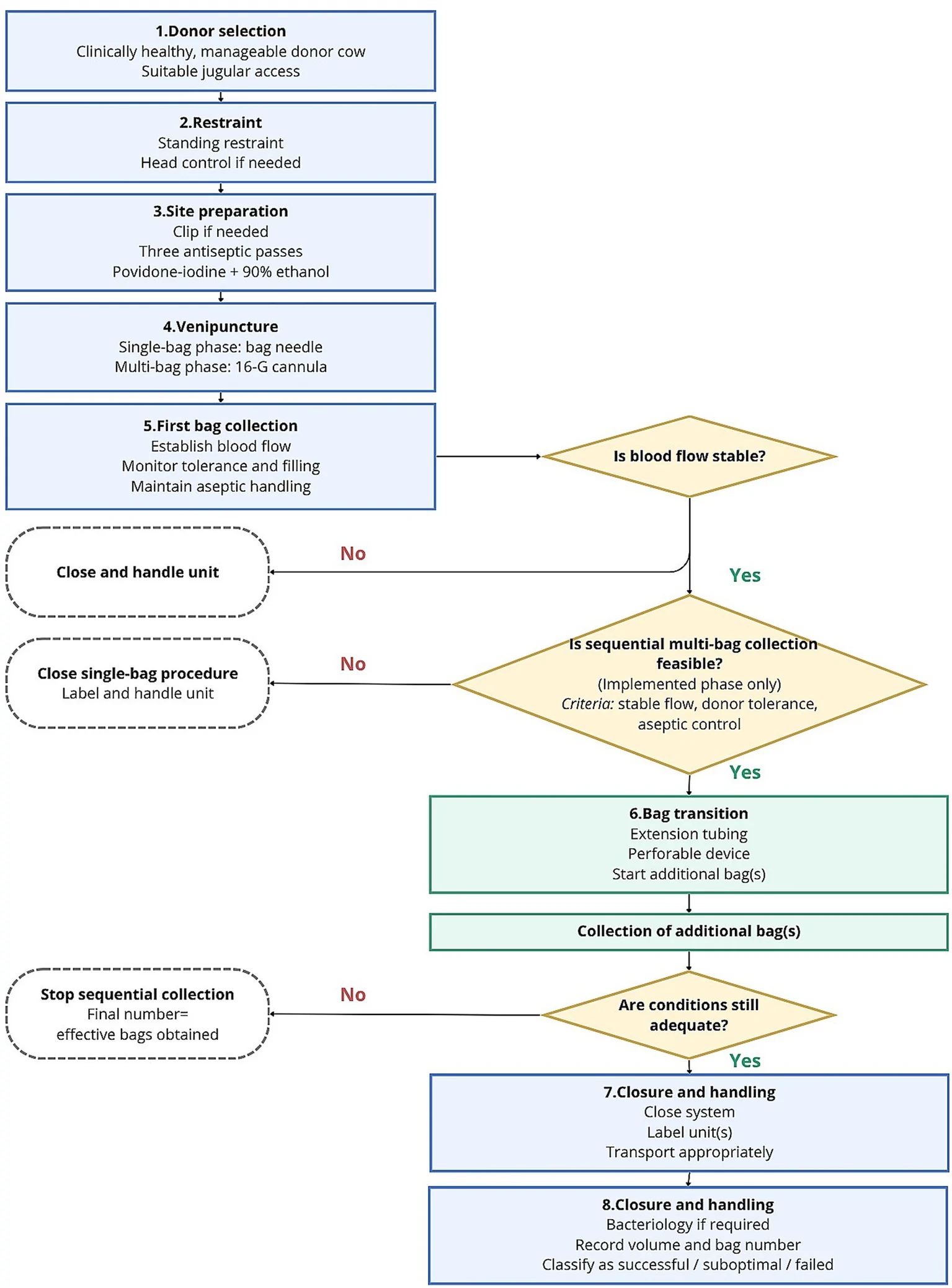
Step-by-step workflow of the large-volume bag-based whole-blood collection protocol in donor dairy cows. The diagram summarizes donor selection, restraint, site preparation, venipuncture, blood flow management, single-bag collection, optional transition to the implemented multi-bag setup, and post-collection handling. Sequential multi-bag collection was introduced only in the later phase of protocol development and was attempted only when blood flow stability, donor tolerance, and aseptic control remained satisfactory.

The overall workflow of the large-volume whole-blood collection protocol is summarized in Figure 1. The detailed step-by-step procedure, including operational setting, timing, critical points, and frequent problems, is reported below.

#### 3.3.1 Step 1. Donor selection

Select potential donor cows from commercial dairy herds on the basis of general health status, absence of systemic illness, absence of ongoing pharmacological treatment, and suitability for jugular venipuncture under field conditions. Donor cows should be clinically fit for donation, manageable during restraint, and suitable for blood collection under field conditions. Preliminary eligibility assessment may be based on available herd records, but final suitability should be confirmed on farm immediately before the procedure.

*Where*: herd records and on-farm clinical assessment.

*Timing*: a few minutes per animal for final assessment before collection.

*Critical points*: general health status, treatment history, donor manageability, accessibility of the jugular region.

*Frequent problems*: unsuitable restraint conditions, poor donor compliance, local alterations interfering with venipuncture.

#### 3.3.2 Step 2. Restraint

Restrain the cow in a standing position using routine farm facilities suitable for jugular venipuncture. Use head control and assistant support when necessary to limit movement and improve procedural stability. Confirm adequate restraint before proceeding to site preparation.

*Where*: on farm, at the collection site.

*Timing*: usually a few minutes.

*Critical points*: stable head and neck positioning, minimal movement, operator and animal safety.

*Frequent problems*: donor movement, unstable head position, inadequate restraint.

#### 3.3.3 Step 3. Site preparation

Perform hair removal when needed before aseptic preparation of the venipuncture site. Prepare the site using three consecutive antiseptic passes with povidone-iodine tincture followed by gauze soaked in 90% ethanol. Maintain clean or sterile handling throughout site preparation and collection to minimize the risk of contamination of both the puncture site and the collection system. Once antiseptic preparation has been completed, avoid contact between the prepared site or collection components and non-sterile surfaces. Do not proceed to venipuncture if aseptic conditions are not considered satisfactory.

*Where*: on farm, immediately before venipuncture.

*Timing*: approximately 2–5 min.

*Critical points*: adequate clipping, complete skin preparation, avoidance of recontamination after antisepsis.

*Frequent problems*: incomplete site preparation, contact with non-sterile surfaces after antisepsis, insufficient drying time.

#### 3.3.4 Step 4. Venipuncture

In the initial protocol, perform venipuncture using the standard needle of the sterile CPDA-1 blood collection bag system. In the implemented multi-bag setup, perform venipuncture using a 16-gauge cannula needle. After successful access to the jugular vein, establish blood flow into the collection system while minimizing manipulation of the collection line. Stable venous access should allow immediate and continuous blood flow into the collection system without repeated repositioning or interruption. Throughout this step, maintain the integrity of the closed collection system and avoid unnecessary manipulation of the line. Do not continue if stable venous access cannot be achieved.

*Where*: on farm, at the prepared jugular site.

*Timing*: immediate once stable venous access is achieved.

*Critical points*: stable venous access, immediate blood flow, minimal manipulation.

*Frequent problems*: unstable needle position, poor venous access, loss of flow after initial puncture.

#### 3.3.5 Step 5. Blood flow management and single-bag collection

Once blood flow has been established, handle the collection bag to maintain continuous flow and preserve system sterility. Monitor blood flow, donor tolerance, and progressive bag filling continuously throughout the procedure. Blood flow should be considered adequate when it remains continuous and allows progressive bag filling without repeated repositioning or prolonged interruption. Discontinue the collection if blood flow becomes intermittent or insufficient to ensure progressive bag filling, if donor compliance decreases, or if any condition arises that may compromise animal safety, sterility, or sample quality. Under standard single-bag conditions, complete the collection when the target volume for one bag has been reached.

*Where*: on farm, during active collection.

*Timing*: variable according to blood flow and donor tolerance.

*Critical points*: continuous flow, prevention of underfilling, donor stability, maintenance of sterility.

*Frequent problems*: slow flow, interruption of flow, donor movement, incomplete filling.

#### 3.3.6 Step 6. Bag transition in the implemented multi-bag setup

Beginning on 24 October 2022, true sequential multi-bag collection became possible using a 16-gauge cannula needle, extension tubing, and a perforable connection device. In this setup, the original bag needle was used to connect the blood collection bag to the extension tubing through the perforable device. Transition to an additional bag was performed while maintaining closed-system handling and minimizing additional manipulation. Proceed beyond the first bag only if all the following conditions are met: blood flow remains stable, donor tolerance is satisfactory, procedural control is maintained, and aseptic conditions are preserved.

Do not proceed to an additional bag if continuation is likely to compromise sterility, fill adequacy, or procedural safety. Sequential multi-bag collection should therefore be considered an optional extension of the protocol rather than an automatic continuation of the first collection step.

*Where*: on farm, during the same collection procedure.

*Timing*: only after satisfactory completion of the first collection step.

*Critical points*: maintenance of flow, secure connection of components, preservation of sterility, realistic assessment of whether continuation is appropriate.

*Frequent problems*: unstable flow during transition, underfilling of additional bags, increased manipulation, sterility concerns.

*Pause point*: no pause is allowed once active blood flow has been established. Transition to an additional bag should be performed only as a controlled continuation of the same procedure.

#### 3.3.7 Step 7. Closure, labeling, and post-collection handling

At the end of the procedure, close the collection system immediately and identify each blood unit without delay. Inspect collected bags visually for obvious abnormalities and handle them under clean conditions to preserve sample integrity. Place the collected units in a clean, protected container and manage them in a way that minimizes contamination and excessive heat exposure during transport. When post-collection bacteriological testing is required, perform sampling using sterile materials and minimize additional manipulation of the closed system.

*Where*: on farm, immediately after collection.

*Timing*: immediately after closure of the collection system.

*Critical points*: correct identification, minimal post-collection manipulation, avoidance of secondary contamination.

*Frequent problems*: labeling errors, delayed handling, contamination during post-collection sampling.

### 3.4 Timing and critical control points

The overall duration of the procedure depends on donor handling, venous access, blood flow stability, and whether sequential multi-bag collection is attempted. Site preparation generally requires a few minutes, whereas the collection phase itself is variable. No pause is allowed after successful venipuncture and establishment of blood flow. In the implemented multi-bag setup, transition to an additional bag should be performed only if blood flow, donor tolerance, and aseptic control remain satisfactory.

The main critical control points in the protocol are donor suitability, adequacy of restraint, proper site preparation, stable jugular venipuncture, maintenance of continuous blood flow, limited handling of the collection system, and strict aseptic technique throughout the procedure.

### 3.5 Troubleshooting

The main technical problems encountered during protocol execution, their likely causes, and the corresponding corrective actions are summarized in Table 2.

**Table 2:** Troubleshooting guide for the large-volume whole-blood collection protocol in donor dairy cows.

Step	Problem	Possible cause	Suggested action
Donor selection	Unsuitable donor	Poor general health status, ongoing treatment, inadequate donor manageability, unsuitable jugular access	Exclude the animal from the procedure and select an alternative donor
Restraint	Inadequate restraint	Donor movement, unstable head position, insufficient assistant support	Improve restraint and head control before proceeding with site preparation or venipuncture
Site preparation	Contamination risk	Inadequate clipping, incomplete antiseptic preparation, recontamination after site preparation	Repeat site preparation if aseptic conditions are compromised before venipuncture
Venipuncture	Difficult venous access	Poor jugular stabilization, local anatomical difficulty, donor movement	Improve restraint and head control, reassess the puncture site, and repeat venipuncture only if aseptic conditions are maintained
Venipuncture / Blood flow management	Low or interrupted blood flow	Unstable needle or cannula position, partial loss of venous access, donor movement	Reposition the needle or cannula when possible; if stable flow cannot be restored, discontinue the procedure
Blood flow management and single-bag collection	Underfilling of the bag	Slow blood flow, early donor intolerance, unstable venipuncture	Complete the collection only if adequate filling can be maintained; otherwise discontinue the collection and classify the procedure according to bacteriological outcome and final volume
Bag transition in the implemented multi-bag setup	Unstable transition to additional bag	Poor flow stability, excessive manipulation, inadequate donor compliance	Do not proceed to the next bag; complete the procedure as a single-bag collection
Bag transition / Additional bag collection	Inadequate fill of additional bag(s)	Continuation attempted despite marginal flow or donor intolerance	Stop sequential collection and evaluate the procedure according to the actual number of effective bags and the final volume obtained per bag
Post-collection handling	Labeling or handling error	Disorganized post-collection workflow, inadequate protection of the unit during transport	Label immediately after closure, place the unit in a clean, protected container, and verify traceability before transport
Post-collection sampling	Secondary contamination during sampling	Non-sterile sampling technique, excessive handling after collection	Perform post-collection sampling with sterile materials and minimize manipulation of the closed system

The table summarizes the main technical problems that may occur during the different steps of the protocol, together with their likely causes and suggested corrective actions.

### 3.6 Post-collection handling and bacteriological assessment

Microbiological assessment was included as part of protocol validation. For each processed blood bag, bacteriological examination was performed on a 10 μL sample collected from the bag at the time of processing for platelet lysate (PL) production. Under a laminar flow hood, the sample was inoculated onto the following media: two blood agar plates, one MacConkey agar plate, one Pseudomonas Agar Base (PAB) plate, one tube of Cation-Adjusted Mueller–Hinton Broth (CAMHB), and one tube of Cooked Meat Medium (CMM).

The inoculated media were incubated at 37 °C for 18–24 h under different atmospheric conditions as follows: MacConkey agar and CAMHB under aerobic conditions; one blood agar plate under microaerophilic conditions (5% CO₂); and PAB, the second blood agar plate, and CMM broth under anaerobic conditions. After 24 h, the culture media were examined for bacterial growth, and subcultures were performed when required. A unit was considered bacteriologically negative when no microbial growth was detected after 5 days of incubation. Post-collection sampling was performed from the collected blood bags, whereas bacteriological examination itself was carried out in the laboratory.

### 3.7 Protocol validation and outcome measures

Because the study encompassed iterative protocol refinement, validation should be interpreted as reflecting performance across successive procedural refinements rather than application of a single unchanged protocol from the outset.

Protocol validation covered four main domains: (i) volume adequacy, based on a predefined threshold of at least 400 mL per bag; (ii) microbiological safety, assessed by bacteriological examination of collected units; (iii) expected performance and repeatability, assessed through the distribution and variability of collected volumes; and (iv) robustness under field conditions, assessed through temporal analyses, protocol-phase comparisons, and exploratory herd-level evaluation.

The main failure modes were microbiological contamination and insufficient bag filling. The 400 mL threshold was selected as a pragmatic operational criterion because it represented both a reasonably filled blood bag in relation to the anticoagulant volume already present in the collection system and a practically useful minimum volume for subsequent processing within the study workflow. This criterion was particularly relevant during the single-bag phase, when the main procedural objective was to obtain at least one adequately filled unit.

Procedural success was defined as obtaining a bacteriologically negative blood unit with a collected volume of at least 400 mL per bag. Procedures yielding bacteriologically negative units with a collected volume below 400 mL per bag were classified as suboptimal. Procedures yielding bacteriologically positive units were classified as failures, regardless of collected volume.

For outcome classification, the adequacy threshold was applied according to the actual procedural configuration. Procedures performed before implementation of the sequential multi-bag setup were analytically treated as single-bag procedures. Procedures performed on or after 24 October 2022 were evaluated according to the number of effective bags obtained within the same collection procedure.

Secondary outcome measures included total collected volume, bacteriological outcome considered separately, number of effective bags obtained per procedure, and temporal changes in collection performance during protocol refinement.

### 3.8 Statistical analysis

The statistical analysis was designed primarily to support method validation by describing protocol performance, variability, and robustness under field conditions rather than testing aetiological hypotheses. Accordingly, descriptive estimates of procedural outcome, collected volume, and microbiological performance were considered the main indicators of method performance, whereas hypothesis-testing results were used as supporting analyses. The following variables were recorded for protocol validation: herd of origin, collection date, protocol configuration, number of effective bags obtained, total collected volume, and bacteriological outcome.

Descriptive statistics were used to summarize protocol performance. Continuous variables were assessed for distribution and reported as median and interquartile range (IQR) or mean and range, as appropriate. Categorical variables were summarized as counts and percentages.

For comparative purposes, procedures were grouped according to protocol configuration into a single-bag phase and an implemented multi-bag phase, the latter beginning on 24 October 2022. Comparisons between protocol configurations were used to describe differences in expected collection output and microbiological performance after implementation of the sequential multi-bag setup. Collected volume was compared using the Mann–Whitney U test. Procedural outcome class (successful, suboptimal, or failed) was compared using the chi-square test, and bacteriological outcome (positive or negative) was compared using Fisher’s exact test.

To explore operator-associated improvement independently of the later protocol modification, additional analyses were performed within the single-bag phase using collection date as a continuous variable. These analyses were intended to assess whether repeated application of the protocol was associated with changes in microbiological reliability or expected collection output over time. The association between collection date and procedural success was assessed using binary logistic regression after dichotomizing procedural outcome as successful versus non-successful (suboptimal or failed). The association between collection date and bacteriological outcome (positive or negative) was also assessed using binary logistic regression. The relationship between collection date and collected volume was evaluated using Spearman’s rank correlation coefficient.

An exploratory herd-level analysis was also performed to assess variability in protocol performance across field settings. Differences in collected volume among herds were assessed using the Kruskal–Wallis test, whereas differences in bacteriological outcome were assessed using the chi-square test. Because herd distribution was unbalanced and partially overlapped with temporal protocol refinement, these analyses were considered exploratory and interpreted cautiously.

Because the collection protocol changed during the study period, analyses involving collected volume were interpreted in light of protocol refinement and effective bag number, which were both treated as process-related variables. Procedures performed before 24 October 2022 were analytically considered single-bag procedures, even when more than one bag was recorded, because these represented separate collections performed in the same donor rather than a true multi-bag configuration within a single procedure.

A two-sided *p* value < 0.05 was considered statistically significant.

### 3.9 Ethics statement

All procedures involving animals were reviewed by the Organismo Preposto al Benessere degli Animali (OPBA) of the University of Padua, which issued a favorable opinion for the study (Project No. 36/2021; Prot. No. 148294; 20 September 2021). The study was classified as a clinical trial outside the scope of Italian Legislative Decree 26/2014. All procedures were performed under field conditions in accordance with applicable animal welfare regulations.

## 4 Method validation results

The following results describe validation performance observed during progressive refinement of the collection procedure, including the later implementation of the sequential multi-bag setup.

### 4.1 Overall procedural success

A total of 294 whole-blood collection procedures performed in donor dairy cows were included in the validation dataset. Based on the predefined validation criteria, 228/294 procedures (77.6%) were classified as successful, 48/294 (16.3%) as suboptimal, and 18/294 (6.1%) as failed. Overall, 276/294 procedures (93.9%) yielded a bacteriologically negative unit. Failed procedures were defined by bacteriological positivity, whereas suboptimal procedures were bacteriologically negative but did not reach the predefined adequacy threshold.

### 4.2 Volume yield and variability

Overall, the median collected volume per procedure was 410 mL (IQR 400–430; range 124.5–1,280 mL), and the mean volume was 454.8 mL. This distribution indicates that, in the overall dataset, collected volume was centered around the predefined adequacy threshold of 400 mL per bag.

For temporal and procedural comparisons, 253/294 procedures (86.1%) were assigned to the single-bag phase, whereas 41/294 procedures (13.9%) belonged to the implemented multi-bag phase, which began on 24 October 2022. During the single-bag phase, the median collected volume was 410 mL (IQR 400–415). During the implemented multi-bag phase, the median collected volume was 872.5 mL (IQR 512.5–897.5). Collected volume was higher during the implemented multi-bag phase than during the single-bag phase (Mann–Whitney U test, *p* < 0.001).

When analytical bag configuration was considered, 263/294 procedures (89.5%) were treated as single-bag procedures, 30/294 (10.2%) as true two-bag procedures, and 1/294 (0.3%) as a true three-bag procedure. Procedures performed before 24 October 2022 were analytically considered single-bag procedures even when more than one bag had been recorded, because these represented separate collections in the same donor rather than a sequential multi-bag configuration within a single procedure.

Under standard single-bag conditions, collected volume was centered around the predefined adequacy threshold for one unit. Later implementation of the sequential multi-bag setup was associated with a higher total volume per procedure.

### 4.3 Microbiological performance

Overall, 18/294 procedures (6.1%) were classified as failures because of bacteriological positivity. All bacteriologically positive results occurred during the single-bag phase, whereas no bacteriological failures were recorded during the implemented multi-bag phase. No statistically significant difference was observed between protocol configurations (Fisher’s exact test, *p* = 0.087). Overall, most procedures yielded bacteriologically negative units.

### 4.4 Operator-associated improvement

To assess temporal changes independently of the later protocol modification, additional analyses were performed within the single-bag phase using collection date as a continuous variable. Within this phase, later collection dates were associated with a lower probability of bacteriological positivity (binary logistic regression, *p* = 0.003).

No significant association was observed between collection date and procedural success when outcome was dichotomized as successful versus non-successful (binary logistic regression, *p* = 0.191). Likewise, collected volume did not show a significant monotonic association with collection date within the single-bag phase (Spearman’s rho = −0.121, *p* = 0.055).

These analyses were intended to explore operator-associated learning effects independently of the later procedural modification. Overall, they suggest improved microbiological reliability over time, whereas volume yield in the single-bag configuration remained comparatively stable.

### 4.5 Performance of the sequential multi-bag setup

During the single-bag phase, 197/253 procedures (77.9%) were classified as successful, 38/253 (15.0%) as suboptimal, and 18/253 (7.1%) as failed. During the implemented multi-bag phase, 31/41 procedures (75.6%) were successful and 10/41 (24.4%) were suboptimal, whereas no failures were recorded. The distribution of procedural outcome classes did not differ significantly between protocol configurations (chi-square test, *p* = 0.089).

Later implementation of the multi-bag setup was associated with increased collection capacity. Because procedural adequacy remained defined on a per-bag basis, higher total volume did not automatically correspond to procedural success, and underfilling of additional bags remained a relevant source of suboptimal outcomes. However, because the multi-bag phase occurred later in the study, this finding should be interpreted cautiously, as the observed difference may reflect both the modified collection setup and accumulated operator experience.

### 4.6 Sources of variability and main limitations

In exploratory analyses, collected volume differed significantly across herds (Kruskal–Wallis test, *p* < 0.001). Bacteriological outcome did not show a statistically significant association with herd (chi-square test, *p* = 0.137).

Some variability in protocol performance should be expected across farms and collection settings. The main causes of non-successful outcomes were insufficient filling of the collection unit, resulting in suboptimal classification, and bacteriological contamination, resulting in failure.

## 5 Discussion

Field validation results indicate that the proposed method, as progressively refined during the study period, provides a reproducible and operationally stable approach for large-volume whole-blood collection in donor dairy cows under commercial farm conditions. Overall, the protocol yielded a high proportion of bacteriologically negative units, and most procedures were classified as successful, indicating acceptable volumetric and microbiological performance. This is relevant because the veterinary literature on blood-derived products continues to highlight incomplete standardization of both upstream and downstream phases, a major source of methodological heterogeneity (6–8).

A first important point concerns the performance of the original single-bag configuration, which should be considered the core operational form of the method. This configuration provided a repeatable volume range centered around the predefined adequacy threshold for one bag, indicating that the protocol can reproducibly yield a collection unit suitable for downstream use when donor handling, venous access, blood flow stability, and aseptic control are maintained. The 400 mL threshold used to define procedural adequacy should be interpreted as a workflow-based operational criterion rather than as a universal physiological standard for bovine blood donation. In the present study, this threshold was considered appropriate because it reflected a reasonably filled bag relative to its anticoagulant content and provided a practically useful minimum volume for subsequent processing. This was particularly important during the single-bag phase, when the main objective was to obtain at least one adequately filled and usable unit.

Although whole-blood collection in cattle is described in the veterinary literature, available references are mainly oriented toward transfusion practice, blood handling, and clinical use rather than toward formal validation of donor-oriented collection procedures (3–5). The present work therefore provides validation data on a collection protocol rather than an emergency transfusion procedure. Its main contribution is not the demonstration of superiority over human or equine collection systems, which was beyond the scope of this study, but the validation of a practical procedure specifically adapted to donor dairy cows under commercial farm conditions. In the present study, “large-volume” should be interpreted as a practical descriptor of collection output rather than as a physiological definition based on a fixed percentage of total blood volume. The term refers to the ability of the method to obtain at least one adequately filled bag and, under suitable conditions, to extend collection sequentially within the same procedure.

A second relevant point is that the protocol improved primarily in microbiological reliability rather than in volume yield with repeated use. Within the single-bag phase, later collection dates were associated with lower bacteriological positivity, whereas collected volume remained comparatively stable. This suggests that operator familiarization mainly improves aseptic consistency rather than changing the expected volumetric performance of the method. From a practical perspective, the protocol therefore benefits from training and procedural stabilization chiefly at the level of microbiological safety.

The implemented multi-bag phase introduced a different type of procedural refinement. The addition of a 16-gauge cannula needle, extension tubing, and a perforable connection device enabled true sequential collection of more than one bag within the same procedure. This later phase was associated with a higher total volume per collection. However, this increase should be interpreted as a structural effect of the modified collection setup rather than as evidence of improved donor-related collection capacity. Accordingly, the number of effective bags is best regarded as a process-related descriptor of method implementation rather than as an intrinsic donor factor. Because the multi-bag phase was implemented later in the study, the apparent improvement in performance cannot be interpreted independently of accumulated operator experience. This later phase should therefore be understood as part of the iterative optimization of the method rather than as the performance of an unchanged protocol applied from the outset.

Importantly, the sequential multi-bag setup should be regarded as a conditional extension of the method rather than its default configuration. Higher total volume did not automatically correspond to procedural success because adequacy remained defined on a per-bag basis. The later-phase data therefore support selective use of this configuration only when procedural conditions remain favorable. When appropriately applied **to** suitable donors, increased collection capacity within a single procedure may reduce the need for repeated blood collection sessions and thereby improve donor management.

Another favorable aspect of the method is that expanded collection capacity did not appear to occur at the expense of microbiological integrity. No bacteriological failures were recorded during the implemented multi-bag phase, although this observation should be interpreted cautiously because of the smaller number of procedures in the later phase. The available data do not allow direct attribution of contamination to a specific procedural step. However, under field conditions, the most plausible contributors include incomplete site preparation, instability of venipuncture technique, manipulation of the collection system, and post-collection sampling. The fact that all bacteriologically positive results occurred during the single-bag phase, together with the temporal reduction in positivity observed within that phase, suggests that progressive procedural stabilization and operator familiarization likely played an important role in improving microbiological reliability over time.

This is particularly relevant in bovine medicine, where platelet concentrates and related hemoderivatives have been investigated for mammary and other clinical conditions, but the available evidence remains limited and methodologically heterogeneous (8–10). In such workflows, the starting whole-blood unit must be not only available in adequate volume but also sterile and anticoagulated to be suitable for downstream processing.

Some variability in performance should be expected across farms and collection settings. The exploratory herd-level analysis suggests that collected volume may vary across herds, likely reflecting differences in handling conditions, donor compliance, and operational context rather than intrinsic limitations of the method itself. The protocol was developed under commercial dairy farm conditions in Italy and was applied by trained personnel. Its performance may therefore differ under other management systems, in different breeds, or when the procedure is performed by operators with less experience. Nevertheless, the core procedural principles—restrained jugular collection, aseptic handling, closed-system management, and selective extension to sequential multi-bag collection—are potentially transferable beyond the specific study setting. More broadly, upstream whole-blood collection may represent a practical limiting step in the development of blood-derived product workflows in bovine medicine. A reliable field method for collecting sterile blood units may support such workflows, whereas poor standardization of the collection phase may hinder their development and evaluation.

Taken together, the validation data support three main characteristics of the proposed method. Consistent with the methodological aim of the study, the descriptive estimates of procedural success, microbiological negativity, and collection output obtained under field conditions represent the main validation outcomes, while formal statistical comparisons should be interpreted primarily as supporting analyses. First, the single-bag configuration provides the most reproducible and operationally stable form of the procedure. Second, microbiological performance appeared to improve with operator experience, emphasizing the importance of training and consistency in aseptic handling. Third, the method is scalable through a sequential multi-bag setup, provided that this extension is applied selectively and only when procedural control remains satisfactory.

The present study also helps define when the method is less likely to perform optimally. Suboptimal outcomes were mainly associated with insufficient filling of the collection unit, whereas failed outcomes reflected microbiological contamination. In practical terms, the method is less likely to perform well when restraint is inadequate, jugular access is unstable, blood flow becomes intermittent, or aseptic handling cannot be maintained. Under such conditions, early termination of the procedure is preferable to forced continuation, particularly when considering progression to additional bags.

This study has some limitations. It was conducted under commercial farm conditions, where procedural uniformity could not be ensured to the same extent as in a controlled experimental setting. The implemented multi-bag phase represented a smaller proportion of the dataset, limiting the strength of some comparisons. Collection time was not systematically recorded during field procedures, which prevented Formal analysis of this operational variable. Finally, direct comparison with previous literature remains difficult because most veterinary publications focus on transfusion use, blood storage, or downstream product preparation rather than on detailed validation of the bovine whole-blood collection step itself (4–8).

In conclusion, the large-volume bag-based protocol described here proved feasible for whole-blood collection in donor dairy cows under commercial farm conditions. The single-bag configuration provided a reproducible and operationally stable basis for collection, while progressive operator experience was associated with improved microbiological safety over time. Subsequent implementation of a sequential multi-bag setup was associated with expanded collection capacity without apparent loss of microbiological reliability, although this later-phase performance should be interpreted in light of both procedural refinement and accumulated operator experience. Overall, the method provides a practical, scalable, and potentially transferable approach for obtaining sterile anticoagulated whole-blood units that may serve as an upstream basis for subsequent processing into blood-derived products.

## Data Availability

The data supporting the findings of this study are available from the corresponding author upon reasonable request. Restrictions apply to public availability because the dataset includes information derived from privately owned commercial farms. Requests to access the datasets should be directed to filippo.rigo@phd.unipd.it.
